# Appearance of the weight-bearing lateral radiograph in retrocalcaneal bursitis

**DOI:** 10.3109/17453674.2010.487245

**Published:** 2010-05-21

**Authors:** Maayke N van Sterkenburg, Bart Muller, Mario Maas, Inger N Sierevelt, C Niek van Dijk

**Affiliations:** ^1^Department of Orthopaedic Surgery; ^2^Department of Radiology, Academic Medical Center, University of Amsterdamthe Netherlands

## Abstract

**Background and purpose:**

A retrocalcaneal bursitis is caused by repetitive impingement of the bursa between the Achilles tendon and the posterosuperior calcaneus. The bursa is situated in the posteroinferior corner of Kager's triangle (retrocalcaneal recess), which is a radiolucency with sharp borders on the lateral radiograph of the ankle. If there is inflammation, the fluid-filled bursa is less radiolucent, making it difficult to delineate the retrocalcaneal recess. We assessed whether the radiographic appearance of the retrocalcaneal recess on plain digital (filmless) radiographs could be used in the diagnosis of a retrocalcaneal bursitis.

**Methods:**

Whether or not there was obliteration of the retrocalcaneal recess (yes/no) on 74 digital weight-bearing lateral radiographs of the ankle was independently assessed by 2 observers. The radiographs were from 24 patients (25 heels) with retrocalcaneal bursitis (confirmed on endoscopic calcaneoplasty); the control group consisted of 50 patients (59 heels).

**Results:**

The sensitivity of the test was 83% for observer 1 and 79% for observer 2. Specificity was 100% and 98%, respectively. The kappa value of the interobserver reliability test was 0.86. For observer 1, intraobserver reliability was 0.96 and for observer 2 it was 0.92.

**Interpretation:**

On digital weight-bearing lateral radiographs of a retrocalcaneal bursitis, the retrocalcaneal recess has a typical appearance.

## Introduction

A symptomatic inflammation of the retrocalcaneal bursa is caused by repetitive impingement of the bursa between the anterior aspect of the Achilles tendon and a bony posterosuperior calcaneal prominence (Stephens 1994, Sofka et al. 2006, DeVries et al. 2009). Most publications have focused on this protrusion, and some have described a loss of radiolucency in Kager's triangle on lateral radiographs. Ultrasonography or MRI can be used to confirm the diagnosis.

**Figure 1.**
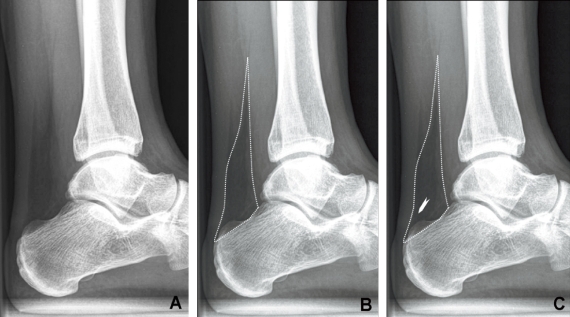
A. Kager's triangle with a normal appearance. B. The triangular lucency with sharp, smoothly curving borders is indicated with the dotted line. C. The arrowhead indicates the retrocalcaneal recess: the “bursal wedge” of Kager's fat pad, which normally forms a radiolucent corner posterosuperior to the calcaneus.

Kager's fat pad, also known as the pre-Achilles fat pad, occupies Kager's triangle (Theobald et al. 2006). Normally this fat pad, as seen on a weight-bearing lateral radiograph of the ankle, is a triangular radiolucency with sharp, gently curving borders (Goodman and Shanser 1997) (Figure 1).

**Figure 2.**
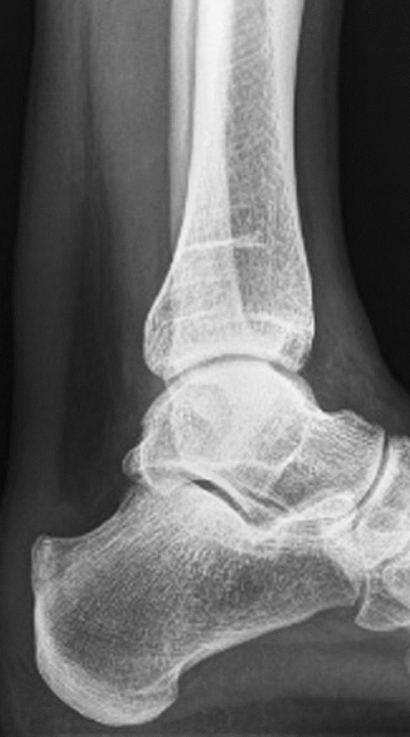
A patient with a chronic retrocalcaneal bursitis. The retrocalcaneal recess has disappeared because of the less radiolucent fluid in the distended retrocalcaneal bursa.

The retrocalcaneal bursa is situated in the posteroinferior corner of the pad. In bursitis, the normally sharply outlined radiolucent retrocalcaneal recess is obliterated (Figure 2). There have, however, been few reports on the appearance of the retrocalcaneal recess on radiography and the series have been small (Pavlov et al. 1982, Heneghan and Pavlov 1984, Heneghan and Wallace 1985). The authors of these articles did not yet have the benefit of digital (film-less) examinations allowing images to be captured, viewed, and reproduced digitally. This offers several advantages since contrast, brightness, and magnification can be adjusted digitally, and a sharper and more detailed view is therefore obtained.

We determined the reliability of the radiographic appearance of the retrocalcaneal recess of Kager's triangle in diagnosing a retrocalcaneal bursitis.

## Patients and methods

**Figure 3.**
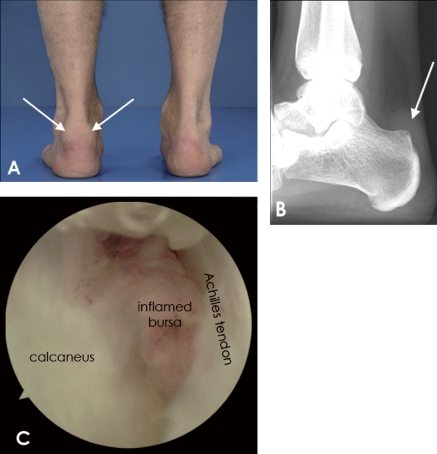
Clinical image (A) and radiograph (B) of a patient with a retrocalcaneal bursitis. The retrocalcaneal recess is obliterated by a chronic inflamed bursa (arrow). C. Endoscopic view of a patient with a retrocalcaneal bursitis.

24 patients (mean age 42 (15–73) years, 25 heels, 13 men) who had digital radiographs taken and who had undergone endoscopic calcaneoplasty for chronic retrocalcaneal bursitis from 2000–2008 were included (Figure 3). 1 patient had had surgery on both ankles on different occasions. Patients with previous hindfoot surgery were not included.

A control group matched for age and sex, consisting of 50 patients (mean age 42 (15–72) years, 59 heels, 30 men) with ankle problems not related to the hindfoot, was identified from our hospital archives from the same period. From the 10 individuals with bilateral radiographs (x of whom were control subjects), we selected 1 heel at random; thus, the study involved 74 heels (from 24 patients and 50 controls). All heels had been examined with lateral weight-bearing radiographs in 20 degrees endorotation, which was the standard procedure at our hospital.

### Observers

To test interobserver reliability, all radiographs were evaluated independently by 2 experienced observers: an orthopedic surgeon and a radiologist. They rated the radiographs positive or negative for the appearance of the retrocalcaneal recess (measurement 1). On a positive radiograph the retrocalcaneal recess of Kager's triangle was less radiolucent, meaning that it was obliterated and had a whiter appearance. On a negative radiograph the retrocalcaneal recess was radiolucent, meaning that it had a black appearance and its borders were sharp. Both observers were blinded regarding the patients' history. For intraobserver reliability testing, both observers evaluated the radiographs 2 weeks later, which were now arranged in a different order (measurement 2).

### Statistics

Descriptive statistics on patient demographics were performed. Sensitivity and specificity were calculated to determine the validity of the test in predicting abnormality of the retrocalcaneal recess of Kager's triangle in retrocalcaneal bursitis. In addition, positive and negative predictive values (PPV and NPV, respectively) were calculated.

For testing of inter- and intraobserver reliability, Cohen's kappa value was used. Kappa values of greater than 0.81 indicate a high association (Sackett 1992). 95% confidence intervals (95% CIs) were calculated for each outcome. We used SPSS for Windows version 15.0 for the analyses.

## Results

We found a high sensitivity and specificity in both measurements for both observers, as well as high PPVs and NPVs (Table 1). The interobserver reliability test for measurements 1 and 2 showed a high association (Table 2). Intraobserver reliability also showed a high association for both observers (Table 3).

**Table 1.** Validity

Observer	1	2
Sensitivity (95% CI)	0.83 (0.58–0.96)	0.79 (0.54–0.93)
Specificity (95% CI)	1 (0.90–1)	0.98 (0.87–1)
PPV (95% CI)	1 (0.75–1)	0.94 (0.68–1)
NPV (95% CI)	0.93 (0.81–0.98)	0.92 (0.79–0.97)

**Table 2.** Interobserver reliability

Radiographs	Kappa (95% CI)
Measurement 1	0.86 (0.71–1)
Measurement 2	0.87 (0.73–1)

**Table 3.** Intraobserver reliability

Radiographs	Kappa (95% CI)
Observer 1	0.96 (0.87–1)
Observer 2	0.92 (0.80–1)

## Discussion

The terminology is confused when describing a retrocalcaneal bursitis, a syndrome of combined bony and soft tissue pathology. Often, Haglund's syndrome, Haglund's disease, Haglund's deformity, pump-bump, and retrocalcaneal bursitis are used interchangeably. To avoid confusion, we chose to use the term “retrocalcaneal bursitis”, which is part of Haglund's syndrome and can be part of Haglund's deformity. The bursal inflammation is the cause of pain and the main reason for treatment.

We have found 2 published studies on the appearance of the retrocalcaneal recess on conventional radiographs. Pavlov et al. (1982) described 10 symptomatic heels and a control group of 78 heels. The goal of their study was to evaluate and describe all available clinical and radiological findings in patients with Haglund's syndrome. The appearance of the retrocalcaneal recess was part of this evaluation. In 1 patient the retrocalcaneal recess was sharp, and in 9 it was ill-defined. Heneghan and Wallace (1985) described the appearance of the retrocalcaneal recess in children. 6 heels were symptomatic, and the control group consisted of 24 heels. The purpose of that study was to describe the osseous and soft tissue findings of retrocalcaneal bursitis and to introduce roentgen criteria to substantiate the diagnosis. The retrocalcaneal recess was one of these criteria. In that study, consistent loss of the sharp definition of the retrocalcaneal recess was also found in symptomatic patients.

We obtained the lateral ankle radiographs during weight bearing and in 20 degrees of endorotation, for parallel alignment of the lateral and medial malleolus and to make sure the lateral and medial aspects of the talus overlapped to be able to see the ankle joint. This is not a standard procedure everywhere, and we do not know whether radiographs obtained in other ways would influence the appearance of the retrocalcaneal recess.

When digital techniques are not available, conventional radiography may still suffice. It is known that lowering the photon energy can be used to clarify details of soft tissues. The results are essentially similar to increasing contrast in the evaluation of a digital radiograph, which has been used in the past to assess patients with Achilles tendon problems (Fischer 1974a,b, Scotti et al. 1979).

In conclusion, the radiographic appearance of the retrocalcaneal recess confirms the clinical diagnosis of a retrocalcaneal bursitis and further diagnostic evaluations are not necessary.
